# The effect of continuous at-home training of minimally invasive surgical skills on skill retention

**DOI:** 10.1007/s00464-022-09277-9

**Published:** 2022-05-23

**Authors:** Maja Joosten, Vera Hillemans, Marije van Capelleveen, Guus M. J. Bökkerink, Daan Verhoeven, Ivo de Blaauw, Bas H. Verhoeven, Sanne M. B. I. Botden

**Affiliations:** 1 Department of Pediatric Surgery, Radboud University Medical Center - Amalia Children’s Hospital, Geert Grooteplein Zuid 10 route 618, Nijmegen, 6500HB The Netherlands; 2 Department of Pediatric Surgery, Princess Maxima Center for Pediatric Oncology, Utrecht, The Netherlands; 3 Department of Surgery, Radboud University Medical Center, Nijmegen, The Netherlands

**Keywords:** Continued training, MIS skills, Simulation-based training, Skill detoriation

## Abstract

**Background:**

Skill deterioration of minimally invasive surgical (MIS) skills may be prevented by continuous training. The aim of this study is to evaluate whether unsupervised continuous at-home training of MIS skills results in better skill retention compared to no training.

**Methods:**

Medical doctors followed a two-week interval training for two MIS tasks (precise peg transfer and interrupted suture with knot tying), ending with a baseline test. They were randomly assigned to the no-practice group or continuous-practice group. The latter practiced unsupervised at home every two weeks during the study period. Skill retention was measured after three and six months on both tasks by the total time needed, distance traveled by instruments and LS-CAT score (8 best possible score and > 40 worst score).

**Results:**

A total of 38 participants were included. No significant differences in performance were found at pre-test or baseline. At six months the no-practice group needed more time for the suturing task (309 s vs. 196 s at baseline, *p* = 0.010) and the LS-CAT score was significantly worse (30 vs. 20 at baseline, *p* < 0.0001). The continuous-practice group performed the suturing task significantly better than the no-practice group at both three and six months (17 vs. 25, *p* < 0.001 and 17 vs. 30, *p* < 0.001) and faster as well (*p* = 0.034 and *p* = 0.001).

**Conclusion:**

This study shows a skill decay after only a few months of non-use and shows better skill retention after continuous unsupervised at-home practice of MIS skills. This indicates an added value of regular at-home practice of surgical skills.

**Supplementary Information:**

The online version contains supplementary material available at 10.1007/s00464-022-09277-9.

Skill retainment of complex procedures remains a problem in current day medicine. Although complex advanced minimally invasive surgical (MIS) skills can be acquired by attending hands-on workshops or courses, these skills will not be retained without sufficient practice [1, 2]. Several studies have reported deterioration of performance of MIS skills over time, while continuous practice on a regular basis prevents this skill deterioration [2, 3]. For complex and rare procedures, sufficient practice in clinical setting is challenging. Simulation-based training might be a solution. Continuous training or warming-up before a procedure using simulation training enhances the performances and reduces operation time effectively [2, 4, 5].

However, training in a skills lab or simulation center often comes with time restrictions. Training is rarely set to fit the trainees’ schedule and is more often set to fit into the simulation centers’ schedule. This may require the trainee to practice when stressed or fatigued [6]. Although voluntary training time is possible in some simulation centers, residents perceive this as inconvenient and not efficient [7, 8]. Furthermore, skills labs are often located at physical distance from the operating theater or the surgical ward, posing a barrier.

Therefore, home-based training of complex skills could be a solution. At-home training has the advantage that it allows trainees to practice more consistently and repetitively when it fits into their own schedule and avoid practicing when feeling fatigued [6]. Simulation training using a take-home box trainer has previously been proven to be associated with improvement in basic MIS skills [9]. For complex MIS procedures it could be used by trainees to acquire surgical skills in the absence of clinical exposure or hands-on courses (such as during the COVID-19 pandemic). Moreover, it may be used to retain the advanced skills needed that were acquired during this period without the opportunity to consolidate these skills in the clinical setting [6].

Although this sounds promising, until today it remains unclear whether at-home practice without expert guidance results in better retention of advanced MIS skills over time. The aim of this study is to evaluate whether continuous at-home training has added value for retention of complex MIS skills.

## Methods

### Study design

The study was designed as an open label randomized trial to evaluate the effects of continuous at-home training on surgical skill retention over time. Written informed consent was obtained from all participants. Approval of the ethics committee of the institution Radboudumc was obtained and approval of the ethical board approval of the ethics committee of Arnhem and Nijmegen was waived. The study was performed in accordance with the ethical standards as laid down in the 1964 Declaration of Helsinki and its later amendments or comparable ethical standards.

### Participants

Senior medical students, medical doctors (not in training to become a surgeon) and PhD students of the Radboudumc, Nijmegen, and University Medical Center Utrecht, Utrecht, the Netherlands were recruited to participate in this study, in the period of May-December 2020. Participants with medical and surgical knowledge and interest but without surgical experience were included. All participants completed an informed consent form and agreed with anonymous processing of the data.

### MIS at-home simulator

**Fig. 1:**
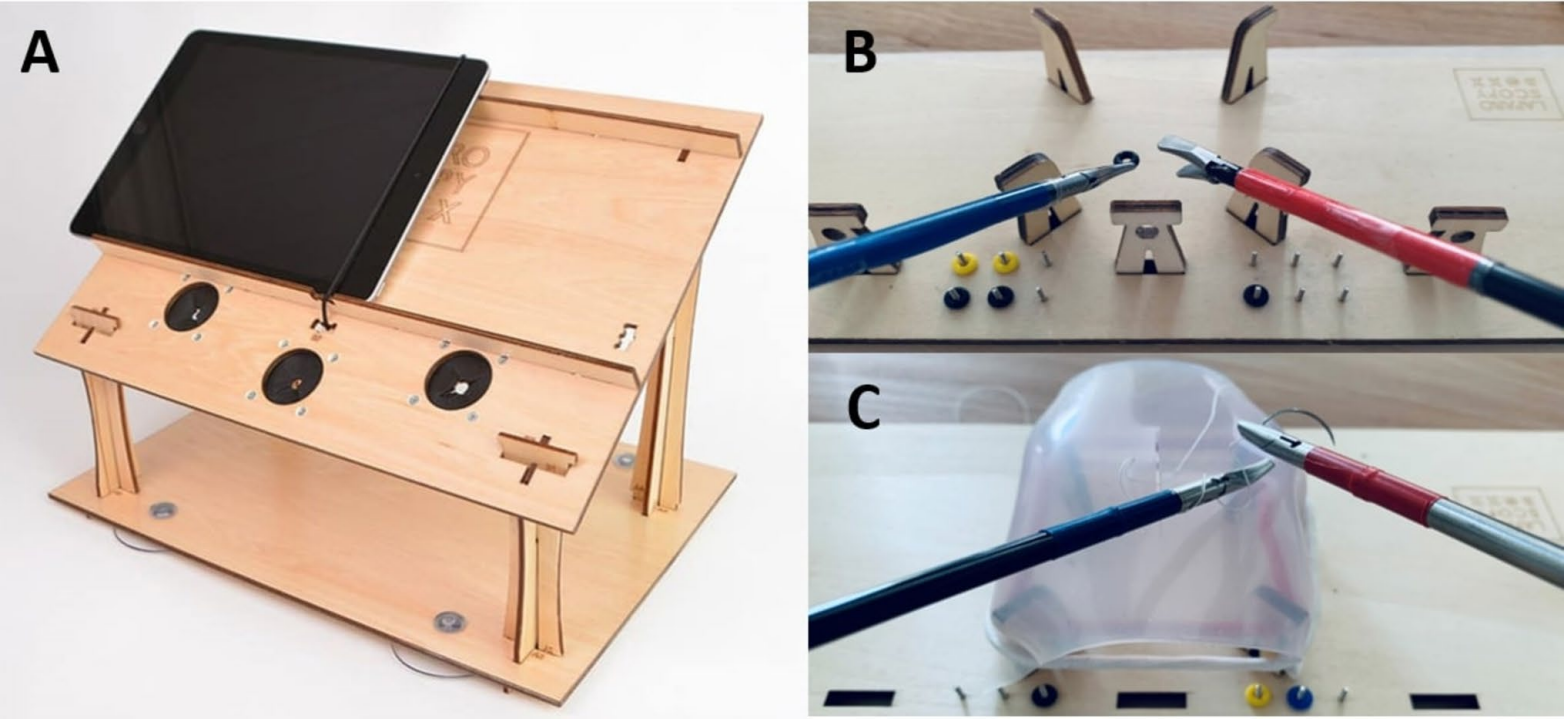
LaparoscopyBoxx take-home simulator used with a tablet (**A**). Task board for precise peg transfer task (**B**) and suturing task (**C**)

The LaparoscopyBoxx is a low budget wooden training box, designed as a build-yourself package (Fig. 1a). This is a take-home MIS trainer, which is easily transported and light weight. The standard version was used in this study. It has three or five instrument ports and has an opening in the center of the top panel, which is designed for the camera of a tablet or a smartphone. In this study, an android based tablet (Lenovo P10) was used with SurgTrac software (Eosurgical ltd., Edinburgh, Scotland, United) to serve as the screen. The instruments used during this study were a 5 mm needle holder, two graspers (one curved and one straight) and scissors.

### Tasks

All participants practiced a precise peg transfer task and a suturing task. The precise peg transfer task required participants to move the six small silicone rings, which were placed on pegs on the left side of the board, to the right side of the board and subsequently back to the left side of the board (Fig. 1b). They had to lift the rings with a grasper in their non-dominant (i.e., left) hand and transfer the rings mid-air to their dominant hand (i.e., right hand). Following, each ring was placed on a peg on the right side of the board. After placement of all six rings on the right side they were transferred to the left side in the same fashion. The maximum time for this task was five minutes.

The suturing task consisted of placing one interrupted suture in a suturing pad (braided 3–0 suture with curved 3/8 17 mm needle) and performing an intracorporeal surgical knot (Fig. 1c). There was no maximum time for this task. Written instructions, a poster with the steps and video instructions on the separate tasks were provided for guidance during training sessions. During all test moments both the precise peg transfer task and suturing task were performed.

### SurgTrac

SurgTrac software, using the application, was installed on the tablet and was used to track the instrument tips [10–14]. This software allows for instrument tracking by means of the colored markings on the instrument tips (red for the dominant hand instrument and blue for the non-dominant hand instrument). The parameters given by this software system for each task were: time needed to perform the procedure (seconds), mean off-screen time for the instruments of the right and left hand (percentage), distance traveled by the instrument tips (meters), workspace (meters), handedness (percentage), speed (m/s), acceleration (m/s^2^), and smoothness (m/s^3^).

### LS-CAT

The competency assessment tool for laparoscopic suturing (LS-CAT) was used for expert assessment of the suturing task. This is a validated assessment tool which consists of two vertical columns representing different task areas and four horizontal rows representing the performance domains, resulting in a total of eight separate items which are scored on a scale of one to four. A lower score indicates a more proficient performance with a score of eight as a perfect score. The third column represents the amount of errors which are scored on the four domains for each task, resulting in 16 separate items (Supplementary Figure S1) [15].

### Outcome parameters

The performance of the suturing task during test sessions was assessed by the parameters distance and time of the SurgTrac software. Previous studies have shown evident construct validity in the parameters time and distance [14, 16] therefore, these are included as primary outcome parameters in this study. The LS-CAT form is used, additionally to the SurgTrac parameters, to provide an objective assessment on the quality of the suture, which is not possible by only measuring time and distance traveled by the instruments. Because the quality of the skills and performed procedure is paramount for a safe and effective treatment, an objective assessment was needed as a primary outcome measurement. All test videos were assessed by a blinded expert observer, who had extensive experience using the LS-CAT form. The performance of the precise peg transfer was assessed by the SurgTrac parameters and additionally the number correctly transferred rings and errors (number of rings dropped) were noted by the researchers, after evaluating each test video.

### Protocol

All participants completed a short questionnaire on demographics and signed an informed consent form. They performed a pre-test to determine their innate abilities. Thereafter they performed an at-home interval training schedule in which they practiced six times 90 min within two weeks. This training schedule was based on previous research showing superiority of interval training over bulk training [17]. Written and video instruction of the two surgical tasks were provided, participants did not receive further guidance during training or test sessions due to the COVID-19 pandemic. After the training program, all participants performed a baseline test. The content of this part of the study was identical for both groups. Participants were then randomly assigned to either the continuous at-home practice group or the no-practice group by a computer algorithm (Castor validated variable block randomization model) [18]. After the baseline test the continuous-practice group continued to practice at home once every two weeks. In each training session they practiced the precise peg transfer task as well as the suturing task and training sessions were at least 30 min long. Training sessions were recorded with SurgTrac to ensure compliance to the protocol. The other group did not practice between test moments. Skill retention was tested after three months and after six months on both tasks, as used at the pre-test and the baseline test (Fig. 4). A flowchart of the study protocol can be seen in Supplementary Figure S2. All training and test sessions were performed at home. All training sessions were performed using the SurgTrac app, which made it possible for the researchers to log every session of the participants and make sure the protocol was adhered. The test sessions were recorded with the camera of the tablet used by the participants and videos were submitted to the researchers. The SurgTrac data was analyzed and videos of the test sessions were assessed on errors and the performance of the suturing task was scored on the LS-CAT by blinded researchers. Blinding of participants was not possible due to the nature of the study.

### Statistical analysis

Data was analyzed by a blinded researcher using IBM SPSS statistics 25 (Armonk, NY: IBM Corp). Differences between the two groups were analyzed with a Mann Whitney U test. Differences in performance between time points were analyzed with a related samples Wilcoxon. Sphericity was assumed if Mauchly’s Test of Sphericity was > 0.05. If sphericity was violated Greenhouse–Geisser was used. A *p*-value of < 0.05 was considered statistically significant.

A sample size calculation was performed with a power of 0.80 and an alpha of 0.05. In order to find a difference of 100 s in total time needed for the suturing task, sixteen participants per group were needed. To account for loss to follow up in this long-term study during the COVID-19 pandemic 20 participants were included in each group.

## Results

### Demographics

A total of 40 participants enrolled in the study. Two participants failed to complete the initial training schedule (one in the continuous-practice group, one in the no-practice group) and were excluded from analysis. Additionally, three participants withdrew consent after the 3 months skill retention test (two in the no-practice group, one in the continuous-practice group). All other participants (n = 38) completed all training- and all test sessions. Mean time between the test moments was 15 days between pre-test and baseline test, 90 days between baseline test and first skill retention test and 88 days between first and second skill retention test. The majority of participants were female (60%), the average age was 25 years and the majority were medical students (66%). There were no significant differences in demographic properties or in surgical experience between the two groups (Table 1).

**Table 1:** Demographic properties of the participants. Values are depicted as mean with standard deviation or number with percentage

Demographics	Total group (*N* = 38)	Continuous practice (*n* = 19)	No practice (*n* = 19)
Age (mean, SD)	25.0 (2.2)	25.4 (2.3)	24.6 (2.1)
Gender (n,%)			
Male	15 (40)	6 (32)	9 (47)
Female	23 (60)	13 (68)	10 (53)
Profession (n,%)			
Medical student	25 (66)	11 (58)	14 (74)
Medical doctor not in training	10 (26)	7 (37)	3 (16)
PhD-candidate	3 (8.0)	1 (5.3)	2 (11)

### Skill regression

Both groups improved significantly between the pre-test and the baseline test for both tasks (*p* < 0.001 for time and distance) as shown in Tables 2 and 3.

**Table 2:** SurgTrac parameters and LS-CAT scores of the suturing task

Suturing task	Randomization group	Pre-test	Baseline	3 Months	6 Months
SurgTrac parameters					
Total time (s)	Continuous practice	661 (499)	218 (102)	221 (73)	189 (90)
	No practice	611 (356)	196 (131)	289 (175)	309 (211)
	*p*-value	0.343	0.343	**0.034**	**0.001**
Distance (m)	Continuous practice	11 (16)	3.9 (6.8)	3.9 (1.8)	3.1 (3.6)
	No practice	10 (8.2)	3.7 (3.0)	10 (15)	6.7 (18)
	*p*-value	0.525	1.00	**0.001**	0.057
LS-CAT scores					
Instrument handling	Continuous practice	15 (1.3)	9.5 (3.0)	7.8 (1.8)	7.3 (2.9)
	No practice	14 (3.1)	9.4 (3.9)	11 (3.7)	13 (2.9)
	*p*-value	0.127	0.977	**0.004**	** < 0.001**
Tissue handling	Continuous practice	15 (1.0)	10 (3.2)	8.4 (1.8)	7.9 (3.5)
	No practice	14 (2.2)	9.4 (4.0)	11 (3.8)	13 (2.7)
	*p*-value	0.075	0.819	**0.008**	** < 0.001**
Errors	Continuous practice	7.1 (3.5)	1.3 (1.0)	0.9 (1.1)	1.3 (1.3)
	No practice	6.0 (4.0)	1.3 (1.5)	2.6 (3.3)	3.8 (3.0)
	*p*-value	0.411	0.968	**0.008**	**0.005**
Total score	Continuous practice	38 (4.9)	20 (6.7)	17 (4.4)	17 (7.0)
	No practice	34 (8.5)	20 (8.6)	25 (9.9)	30 (7.9)
	*p*-value	0.149	0.982	**0.006**	** < 0.001**

Bold values indicate *p *< 0.05

Values are stated as median with interquartile range; ability to perform a knot is stated as number (%). Groups are compared with a Mann Whitney U test

**Table 3:** SurgTrac parameters and objective observed outcomes of the precise peg transfer task

Peg transfer task	Randomization group	Pre-test	Baseline	3 Months	6 Months
SurgTrac parameters					
Total time (s)	Continuous practice	300 (0.0)	148 (57)	143 (64)	149 (67)
	No practice	300 (6.0)	177 (72)	186 (138)	234 (134)
	*p*-value	0.154	0.212	**0.013**	** < 0.001**
Distance (m)	Continuous practice	13.6 (20)	9.1 (12)	6.4 (20)	3.9 (14)
	No practice	15.5 (42)	5.7 (13)	15 (16)	23 (37)
	*p*-value	0.563	0.751	0.130	**0.017**
Observed outcomes					
Total rings (N)	Continuous practice	6.0 (9.0)	12 (0)	12 (0.0)	12 (0.0)
	No practice	7.0 (7.0)	12 (0)	12 (4.0)	12 (1.0)
	*p*-value	0.358	0.799	0.061	0.127
Rings dropped (N)	Continuous practice	(2.0)	0.0 (1.0)	0.0 (1.0)	1.0 (1.0)
	No practice	(4.0)	0.0 (1.0)	1.0 (1.0)	1.0 (2.0)
	*p*-value	0.126	0.343	**0.049**	0.345

Bold values indicate *p *< 0.05

Values are stated as median with interquartile range. Groups are compared with a Mann Whitney U test

**Fig. 2:**
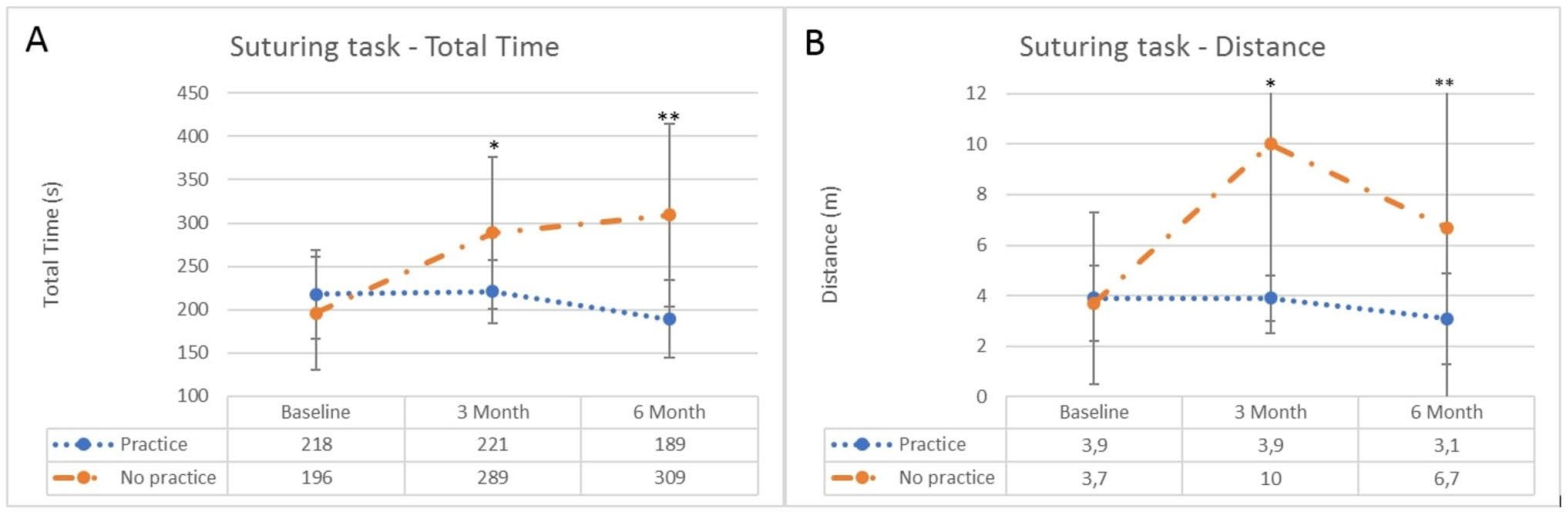
**A** Total time in seconds of the suturing task for the no-practice group and continuous-practice group for the different test moment (baseline test, 3 months, 6 months). *p = 0.034; **p = 0.001. **B** Distance of the suturing task in meters for the no-practice group and continuous-practice group for the different test moment (baseline test, 3 months, 6 months). *p = 0.001

**Fig. 3:**
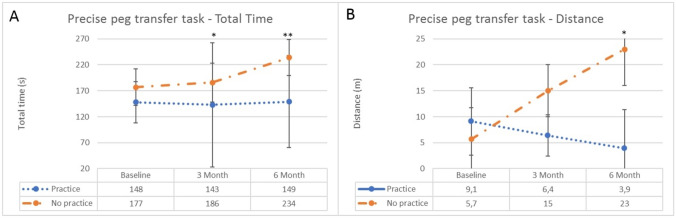
**A** Total time in seconds for the peg transfer task for the no-practice group and continuous-practice group for the different test moment (baseline test, 3 months, 6 months). *p = 0.013, **p < 0.001. **B** Distance of the peg transfer task in meters for the no-practice group and continuous-practice group for the different test moment (baseline test, 3 months, 6 months). *p = 0.017

The continuous-practice group retained their skill level after the baseline test for both the peg transfer and the suturing task. The skill levels of the no-practice group evidently declined after three months and continued to decline at the second skill retention test, as shown in Figs. 2 and 3. The total time needed to complete the suturing task increased from 196 s at baseline to 309 s at six months (*p* = 0.010). The distance of the instrument tips doubled from 3.7 m at baseline to 6.7 m after six months (Table 2). This increase in distance was even more evident for the precise peg transfer task (baseline: median 5.7 m, to six months: median 23 m, *p* = 0.013), as shown in Table 3.

**Fig. 4:**
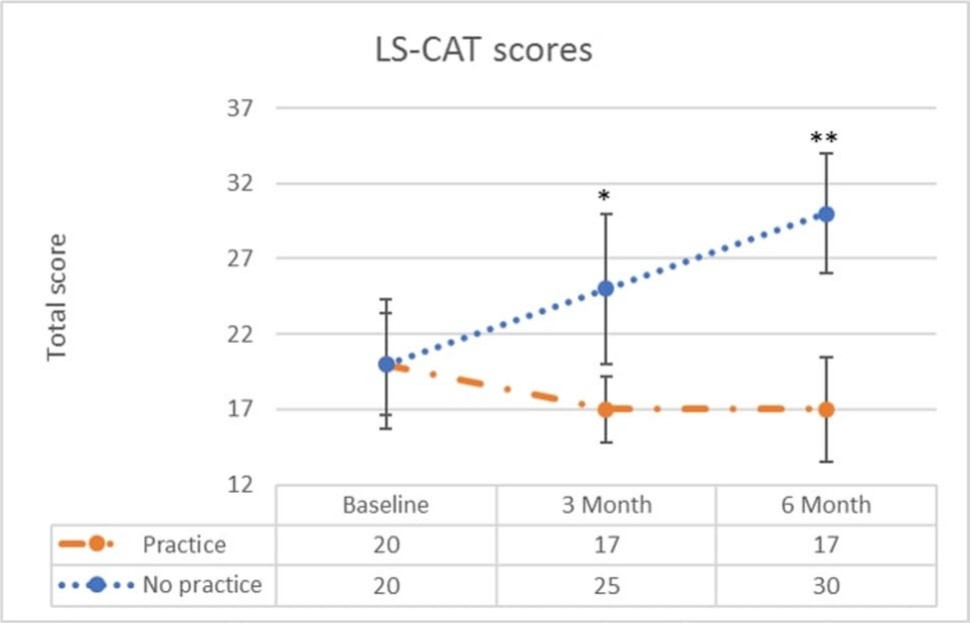
Total scores of LS-CAT for the suturing task for the no-practice group and continuous-practice group for the different test moments (baseline test, 3 months, 6 months). *p = 0.006; **p < 0.001

The decline in skill level was reflected in the LS-CAT scores as well. The total scores increased with 5 points after three months (baseline: 20 vs. 3 months: 25, *p* = 0.039) and 10 points at six months (baseline: 20 vs. 6 months: 30, *p* = 0.002). The number of errors doubled from baseline to three months (mean 1.3 vs. mean 2.6, *p* = 0.275) and tripled at six months (mean 1.3 vs. mean 3.8, *p* = 0.014) (Table 2 and Fig. 4).

### The added value of continuous practice

There were no significant differences at the pre-test and baseline test between the two groups for the suturing task. The continuous-practice group performed significantly better on the suturing task at three and six months (Table 2 and Fig. 2). They completed the suturing task significantly faster at both test moments (median 221 s vs. median 289 s, *p* = 0.034 at three months and median seconds 189 vs. median 309 s, *p* = 0.001 at six months) (Fig. 2a). Additionally, the continuous-practice group demonstrated higher precision and better instrument control, because the distance covered by the instrument tips was at least twice as low in both test moments (median 3.9 m vs. median 10 m, p = 0.001 and median 3.1 m vs. median 6.7 m, *p* = 0.057) (Fig. 2b).

This effect was also seen in the precise peg transfer task (Table 3). After three months, the continuous-practice group was significantly faster (median 143 s vs. median 186 s, *p* = 0.013) and this difference was even larger after six months (median 149 s vs. 234 s, *p* < 0.001) (Fig. 3a). The path length of the instrument tips was shorter in both test moments, however only significantly after 6 months (median 3.9 m vs. median 23 m, *p* = 0.017) (Fig. 3b).

The scores of the LS-CAT reflected this increase in skill after continuous practice as well, with a significant improvement in scores after six months (baseline: 20 vs. 6 months: 17, *p* = 0.042). The continuous-practice group scored significantly better than the no-practice group at three and six months. They demonstrated better instrument handling at three months (mean 7.8 vs. mean 11, *p* = 0.004) and this difference in scores was even larger at six months (mean 7.3 vs. mean 13, *p* < 0.001). Additionally, they demonstrated better tissue handling at both skill retention tests (mean 8.4 vs mean 11, *p* = 0.008 at three months; mean 7.9 vs. mean 13, *p* < 0.001 at six months). Furthermore, the number of errors in the no-practice group was at least twice as high as in the continuous-practice group (mean 0.9 vs. mean 2.6 *p* = 0.008 at three months; mean 1.3 vs. mean 3.8, *p* = 0.005 at six months), as shown in Table 2.

### Warming-up

The majority of the no-practice group participants violated the protocol and practiced the suturing task before the test at three months (*n* = 17) and six months (*n* = 18). This was a significantly higher number of participants compared to the continuous-practice group (*n* = 2, *p* < 0.001 for both test moments). They practiced a maximum of six times before the suturing task and a maximum of five times before the precise peg transfer task, with a median of two runs per warming-up per participant. At the pre-test and baseline test only a minority of participants practiced before the suturing task (continuous-practice group: pre-test n = 1 and baseline *n* = 0; no-practice group: pre-test *n* = 3 and baseline *n* = 3).

## Discussion

This study shows that there is an evident decay in MIS skills if these are not practiced regularly. Continuous at-home training can prevent this skill decay and may even result in an increase of MIS skills. This result was not only found for the more complex MIS suturing task, but for a precise peg transfer task as well.

This is the first study to assess MIS skill retention by objective assessment parameters as well as expert assessment, which both showed an evident decay in skills after a period of non-use.

These findings are in line with literature showing that acquired skills will decay over time after periods of non-use, potentially leading to patient unsafety in clinical settings [19]. As known from sports and music literature, focussing on activities that are specifically designed to improve performance (deliberate practice) results in a higher level of proficiency. Expert performance is acquired gradually and improvement in performance requires suitable training tasks for deliberate practice. When proficiency is reached, however, practice is necessary to maintain the same skill level [20, 21]. Previous research revealed that some trainees were not able to perform trained skills to proficiency a mere month after finishing their training [22, 23]. This implies that practicing until proficiency alone is not enough to guarantee long-term skill retention and patient safety. Despite excellent initial training, in the absence of routine clinical use, complex skills -such as suturing- decay, indicating the need for continued training [3].

However, skill decay is not limited to low-volume procedures. A study by Castellvi et al. found that even with adequate case volumes of MIS operations according to the Accreditation council for graduate medical education, ongoing clinical training is insufficient for most residents to maintain their MIS skills [24]. Furthermore, surgical residents may acquire advanced MIS skills at hands-on courses without the opportunity to consolidate these skills in clinical setting afterward [25]. These newly acquired skills will decay without sufficient practice.

During the COVID-19 pandemic, surgical trainees face an even greater challenge in acquiring and retaining surgical skills. In many countries only urgent or emergency surgery is performed and elective surgeries are postponed. These elective cases for benign disease are usually performed by surgical residents under expert guidance or supervision. Furthermore, regulations permitting only essential personnel in the operating room and shortage in personal protective equipment further limit the trainees’ opportunities to attend, observe and assist in surgical procedures [26–28]. Moreover, most hands-on workshops have been canceled, reducing the opportunities for continuous education of trainees even more [29, 30]. Continued unsupervised at-home training could be a solution to this problem, improving these skills and ensuring skill retention by regular practice.

The positive effect of continuous practice might even be underestimated in this study. Despite the fact that the no-practice group was instructed not to practice before or between test moments, the vast majority of the participants ignored this instruction and practiced one or more times before the test. Practicing before a test or procedure is known as warming-up. However, despite the warming-up of the no-practice group, the continuous-practice group scored significantly better at both retention tests for both tasks. This indicates that repeating the exercise moments before the test (warming-up) alone is not as effective for skill retention as structured continued training. Previous research has shown that warming-up has a positive effect on surgeon’s performance during a procedure [31, 32]. This study suggest that continuous training is even more effective than only warming-up before a procedure.

### Future perspectives

We propose continued training of MIS skills after a hands-on course to consolidate the newly acquired skills. Consideration needs to be given to how the trainees can apply these skills in the clinical setting. If training in the clinical setting is not feasible, unsupervised at-home training could prevent skill decay. Course designers could focus on continued training after the course, preferably on take-home simulation models to make at-home training feasible.

In this study the continuous training group practiced once every two weeks. This was based on previous research regarding interval training and on a study by Gallagher et al. who found that surgical skills decline after two weeks of non-use [16, 31]. However, the optimal intersession interval (time between two independent training sessions) for continuous training remains unknown, particularly for the long term. Further research may investigate whether a shorter interval results in even better skill retention over time or that on the long term other intervals are sufficient to retain the skills.

## Limitations

Despite the fact that surgical residents are the target group, they were not included as participants. This was because surgical residents are exposed to more confounders (such as training and workshops on MIS skills and exposure to MIS procedures in the clinical setting) and subsequently introduce more bias in the results. Therefore, participants with surgical knowledge and interest, but without surgical experience, were included.

Motivation to practice MIS skills outside of the work setting may vary among participants. Previous studies by Blackhall et al. and Glostlow et al. have shown that competing commitments and time restrains can pose a barrier for trainees regarding at-home simulation training [33, 34]. However, variances in motivation would mostly be corrected by randomizations. Furthermore, the participants all participated voluntarily and completed all tests and training sessions as dictated by the protocol. Although it was not possible to blind participants, the subsequent risk of bias was regarded low due to the motivation of the participants to perform the tests at their full ability. Furthermore, outcome measures were objective and analysis was performed by a blinded researcher. The majority of participants in the no-practice group did deviate from protocol and practiced before the test session. However, as stated previously, this may result in an underestimation of the positive effect that was found.

## Conclusion

There is an evident decay in MIS skills if these are not practiced on a regular basis. Warming-up alone is not enough to prevent this skill decay. Skill decay can be prevented by continuous at-home training of MIS skills, which may even result in an increase of MIS skills.

## Supplementary Information

Below is the link to the electronic supplementary material.

Supplementary file1 (DOCX 84 kb)

Supplementary file2 (DOCX 90 kb)
