# Supplementary figures and images for: The effect of continuous at-home training of minimally invasive surgical skills on skill retention
Source: Surg Endosc. 2022 May 23;36(11):8307–15. doi: 10.1007/s00464-022-09277-9 (PMC9125971; doi:10.1007/s00464-022-09277-9)

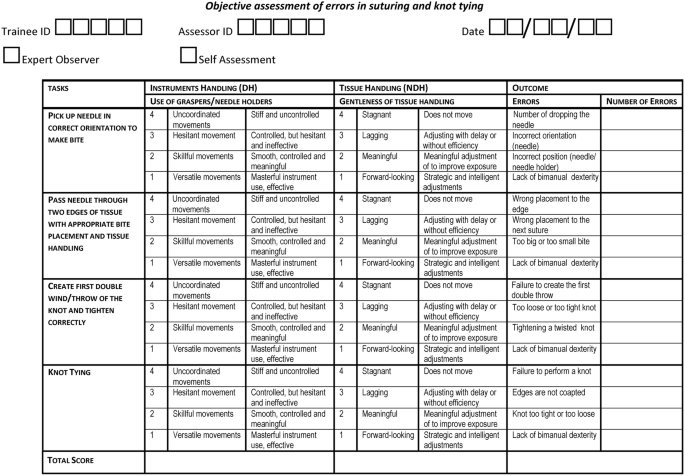


Supplementary figure 1: LS-CAT, a validated tool for assessment of laparoscopic suturing [15].

Supplement: Supplementary file 1 — Supplementary file1 (DOCX 84 kb) [file 464_2022_9277_MOESM1_ESM.docx]

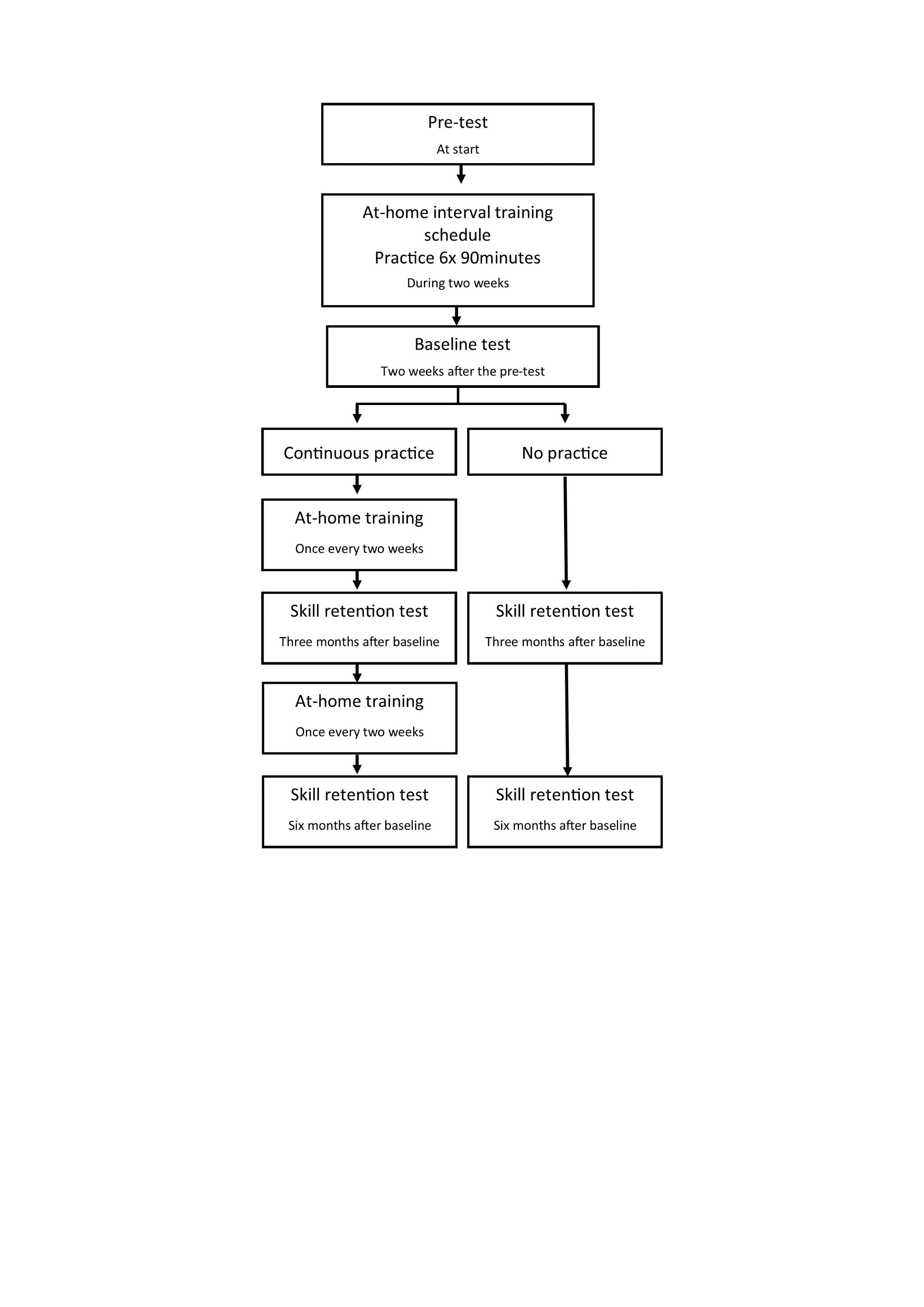


Supplementary figure 2: Flowchart of the study protocol

Supplement: Supplementary file 2 — Supplementary file2 (DOCX 90 kb) [file 464_2022_9277_MOESM2_ESM.docx]
